# The Port-Folio; or, Medical and Surgical Miscellany

**Published:** 1818-10-01

**Authors:** 


### FIFTH DEPARTMENT.

THE

**Port-Folio;**

OR,

MEDICAL AND SURGICAL MISCELLANY.

### I.

### *A Case of Inflammation of the Tongue, and of Sublingual Abscess, producing Symptoms requiring the Operation of Laryngotomy, and terminating favourably.* By Surgeon Hamilton, of the 13th Regiment of Foot.

[Communicated through the Army Medical Board.]

July 25th, 1818. Two o'clock, P. M.—John Herbert, a recruit of the 13th foot, 20 years of age, and of a robust constitution, quartered at Vale Castle, states, that he has felt for the last two days, a sense of swelling and restriction of his tongue; some difficulty in swallowing, and pain in speaking; the tongue appears thickened towards its base, and elevated by sublingual tumefaction nearer the tip; there is also some external swelling under the chin, but no discolouration or heat of skin; the view (rather imperfect) which can be got of the fauces, discovers no inflammation there.

Venesectio ad ℥xxxvi. Capiat pulv. jalapii ʒß; submur. hydrargyri gr. x. The liniment. volatile to be applied externally.

Eight o'clock.—There is no observable change, but the patient thinks himself more at ease. Had one lax stool from the medicine.

To use a nitre gargle.

26th. Eight o'clock, A. M.—Sublingual affection increased; has had no stool since last visit.

Habeat enema comm. statim, et post horam, sulph. magnesiæ ʒvj solutæ in inf. sennæ ℥iij.

Two o'clock, P. M.—Medicine operating freely; yet the symptoms are advancing, his speech is almost unintelligble, and the difficulty of swallowing is augmented. Thirty leeches were now applied over the throat.

July 26. Five o'clock, P. M.—Leech-bites bleeding, and he thinks himself better.

Seven o'clock.—Some blood still flowing from the leech-bites. The patient's countenance has a sunk appearance; pulse slow and compressible; skin clammy; can, with much difficulty, swallow the smallest quantity of fluid, and occasionally makes a convulsive struggle to get breath. The sublingual tumour, now appearing to contain matter, was opened, and from it there was discharged, at once, about half an ounce of bloody pus. The inhaler was now made use of, but with what effect cannot be said.

Half past nine o'clock.—A quarter of an hour ago, the patient's struggles for breath became alarming; he now lies apparently lifeless; no proof of the circulation in the pulsation of any of the arteries; face livid; extremities deathly cold.

While doubtful of the efficacy of the only probable means by which life could be rescued, I had almost said restored, I did not hesitate to perform laryngotomy instantly.

The steps of the operation were as follow:—A lancet being most ready, with the shoulder of this instrument I made an incision about au inch in length, commencing below the thyroid cartilage, and, exposing the cricoid cartilage, introduced the point of the lancet perpendicularly, as close as possible to the upper margin of this last cartilage, thrusting it forward until it entered the larynx; it was then carried towards the thyroid cartilage, in the line of its longitudinal angle, by which an opening into the larynx of a third of an inch was formed: the eye-end of a silver probe, introduced flatways, and when fairly within the orifice, giving it a half turn, procured a sufficient passage for the air. All this was done in, I believe, the course of a minute, and it was not before a second minute elapsed that the patient shewed signs of life in an action of the larynx which forced out the probe; in three minutes he opened his eyes, and appeared sensible, and a little after tossed himself with some force from his back to his side, and immediately returned to the original posture. The probe introduced a second time produced irritation, and was therefore withdrawn; happily, howver, it was not necessary towards keeping the passage free, respiration going on with tolerable ease and regularity.

Ten o'clock.—His countenance has now become natural, as also eat of skin.

Eleven o'clock.—The lingual affection does not subside, as might be expected from the opening of the abscess. The throat, with the exception of the point of operation, and a little way around it, was now covered with a blister.

27th. Six o'clock, A. M.—The patient was restless from twelve to four, probably from the pain of the blister; at this last hour he fell asleep, and still continues in that state.

July 27th. Eight o'clock, A. M.—He has just now awoke, decidedly relieved; the tongue and tumefaction underneath much reduced. The blister rose well. The inhaler appears grateful by his holding it himself; he swallows a little tea, but with pain.

Ten o'clock.—He drinks tea with tolerable ease; the larynx being still, externally, pervious, he has, of course, no voice.

Five o'clock, P. M.—He has taken six ounces of gruel, with two ounces of wine in it, since last visit. The opening into the larynx being now closed, he speaks distinctly, though with a weak voice.

28th.—Slept well; recovering rapidly.

29th.—Closed the wound. He eats bread sopped in his tea. Bowels opened last evening by an enema.

30th.—Continues to do well. From this date to the 30th of August, the day on which he was discharged from hospital, he was progressively changed from spoon to low and half diets, guided by the state of convalescence.

In describing the operation, it should have been observed, that there was no bleeding, and that the thyroid gland was very easily avoided.

HENRY HAMILTON,

Surgeon 13th Foot.

*Guernsey, Sept.* 2, 1818.

☞ The Editor returns sincere thanks to the Army Medical Board, and to Mr. Hamilton, for the communication of the foregoing very interesting Case, while he pledges himself that the pages of the Medico-Chirurgical Journal shall always feel proud to bear the records of Military Medicine and Surgery.

### II.

### *List of Cases admitted under the Care of the Physicians of the Universal Dispensary for Children, St. Andrew's Hill, Doctors' Commons, between April 1, 1818, and September 1, following.*

| Diseases. | No. of Cases. | Diseases. | No. of Cases. |
|---|---|---|---|
| Synocha | 51 | Pertussis | 75 |
| Synochus | 98 | Diarrhœa | 21 |
| Typhus | 21 | Dysenteria | 10 |
| Febris Intermittens | 10 | Tabes Mesenterica | 19 |
| —— Remittens | 8 | Tormina | 8 |
| —— Catarrhalis | 24 | Cynanche Trachealis | 3 |
| Pneumonia | 17 | ——— Parotidea | 2 |

| Diseases. | No. of Cases. | Diseases. | No. of Cases. |
|---|---|---|---|
| Cynanche Tonsillaris | 12 | Dyspepsia | 26 |
| Tussis | 15 | Atrophia | 5 |
| Phrenitis | 14 | Hemiplegia | 4 |
| Ascites | 7 | Scrophula | 7 |
| Cholera Morbus | 34 | Febris Hectica | 10 |
| Morbilli | 49 | Chorea Sancti Viti | 12 |
| Scarlatina Simplex | 6 | Epilepsia | 13 |
| Varicella | 15 | Convulsiones | 26 |
| Paralysis | 4 | Colica | 15 |
| Hydrocephalus Acutus | 28 | Cephalalgia | 7 |
| ——— Chronicus | 29 | Pemphigus | 2 |
| Urticaria | 7 | Valetudo Infirma post Morbillos | 13 |
| Dyspnœa | 6 | Pleurodynia | 4 |
| Icterus | 11 | Epistaxis | 2 |
| Aphthæ | 19 | Vermes Ascarides | 20 |
| Pleuritis | 8 | ——— Lumbrici | 12 |
| Erysipelas | 14 | ——— Tænia | 2 |
| Pompholyx | 6 | Hæmatemesis | 3 |
| Roseola | 7 | Psoriasis | 8 |
| Erythema | 15 | Vomitiones | 9 |
| Hæmoptysis | 3 | | |
| Phthisis | 2 | | |
| Rheumatismus | 14 | Total | 903 |
| Rachitis | 6 | | |
| Enteritis | 23 | | |

From the first to the tenth of April, the cases of Pneumonia brought into the Institution were numerous; but many of them were indistinctly marked in the beginning and the Pyrexia slight: Catarrh was frequently the fore-runner. In other instances, the membrane of the trachea was the primary seat of disease. Pyrexia; short and difficult breathing; a strong and rapid pulse, were the first symptoms; to which succeeded, after five or six days, a painful and violent cough, flushed face, anxiety, restlessness, and all the well marked symptoms of Pneumonia. The other diseases which occurred about this period in the Institution were Measles, Hooping-Cough, and Scarlatina, all of which were extremely severe, and all in turn modified with diarrhœa, and acute affections of the head. A bilious fever, commonly termed bilious remitting fever, prevailed also among children from three to ten years of age. But, it is proper to remark, that the remissions in this fever were generally so slight as scarcely to give a remitting type to the disease, and the more the fever assumed the character of synochus, the plainer it was to see that the functions of the liver were more particulary disturbed than the functions of any of the other abdominal viscera. Still, no malignant symptoms appeared in the progress of the disease; and whatever difference in the character of diseases, in which pyrexia, without topical inflammation, is the predominant symptom, may have formerly existed at certain seasons, in the course of a series of years, even from the time of Sydenham to our own days, no fact in medicine, it may with truth be said, is better ascertained, than that malignant fever in general has neither been so frequent nor so severe, during the last ten or twelve years, as it was antecedent to that period.

Modern physicians, convinced that too slight an attention had formerly been given to the state of the secretions of the alimentary canal, and of the liver, in fever, and aware of the importance of this particular, have judiciously made an alteration in the condition of the secretions in these organs a primary object in their practice. Such an omission as this in the mode of treatment pursued by the old physicians, is, in itself, sufficient to account for the malignancy of the febrile disorders in their days; if not, to what are we to attribute the success now so universally met with in treating fevers precisely of the same natuie; and, in many instances, threatening to become equally severe as in former years? Are we not to conclude, that the success of modern practice is chiefly owing to the bold plan of depletion, by means of purgatives administered at intervals through every stage of malignant fever?

The Measles, in the months of May and June, were attended with very high pyrexia: in no disease did inflammatory action run to a greater height than in measles of this season. A true synocha accompanied the measles during several days; the pulse was hard and strong at 150; the tongue brown and dry; the lips parched; the face flushed; the respiration quick, and often laborious; the skin extremely hot and dry. Even in fevers of a continued type, without topical inflammation; and also in those fevers which accompanied other exanthemata, the pyrexia was never so intense as in measles. In many of the cases of this disease, which had been neglected in the beginning, the tongue, lips, and teeth were covered with the same kind of sordes as in typhus; still, no livid petechiæ appeared upon the body or extremities.

Two cases of Anasarca, after Erysipelas, were admitted into the Institution; they were both of some duration, but ended well. Two cases of Intestinal Hæmorrhage occurred in boys about ten years of age, labouring under Synochus. One of the boys died; the other had a lingering recovery.

A boy, twelve years of age, had Chorea Sancti Viti in its acute form, which proved very tedious and difficult to cure: it appeared to be idiopathic. The digestive organs, contrary to what is usual in this disease, scarcely experienced atony or irritation. The pyrexia was very considerable; the pulse both strong and quick; the head was painful; the pupils were dilated, but the pupil of the right eye was more dilated than the pupil of the left eye; the tongue was white, and covered with a moist tenacious sordes. Almost every part of the head and face, trunk and extremities, was in constant motion; the arms were tossed up and down by violent jerks. The boy could neither stand, sit, nor lie still; but all his motions were more moderate when he was put into an horizontal posture. He was frequently delirious; and, at intervals, answered as an ideot: but, at last, he lost his speech for a fortnight. At times he sighed deeply. A month after the attack the febrile symptoms abated, and the disease put on the chronic character. In this state he continued three weeks, and was then again attacked with severe pyrexia, as at first; and when this once again subsided, the disease persisted under its chronic form for three weeks longer; at the end of which time he began to mend, and was perfectly restored in about four months from the first attack, by a long perseverance in the use of chalybeates, and by changing his residence into the country.

In July and August, Synochus, variously modified with Diarrhœa, Pleuritis, and inflammatory affections of the head, was very prevalent. In general, the stomach also partook, in a high degree, of the disease of the system, tension and pain prevailing in the epigastric region, with a tongue denoting excessive gastric irritation. A few cases only of Typhus Mitior were brought into the Institution during these months, and these cases were all evidently milder than those which fell under our notice in the beginning of the summer. Synocha from cold, Synocha from heat, and symptomatic Synocha were met with throughout the months of July and August. Children, particularly those of two years of age and upwards, were as frequently the subjects of attack of Cholera Morbus as adults. In protracted instances of this disease, a copious hæmorrhage took place, sometimes oftener than once, from the bowels; and Enteritis was no uncommon occurrence after several day's continuance of Cholera Morbus. Accompanied with fever of the character of Synochus, inflammatory affections of the liver, stomach, and other chylopoietic viscera, were frequent; all these were, for the most part, attended with considerable tension of the abdomen, and a peculiar tenderness, such as is met with in peritoneal inflammation. In the bodies of two children, who died in this state, extensive adhesions were found, of the omentum to the peritonæum, and of this membrane to the liver and instestines. Intus-susception was also met with in both, and marks of inflammation in the intestines, particularly in the colon, which was highly inflamed, and in various parts contracted. Several well-marked cases of Erysipelas Infantile, in children about nine weeks old, occurred, and ended favourably. Scarlatina was in general mild, Measles also; but the Hooping-cough, on the contrary, was frequently severe, and combined with Pneumonia, Hydrocephalus, and Paralysis. Catarrhal affections were seldom seen till the latter end of August. Phrenitis and Hydrocephalus Acutus were very common, not only in children about the period of dentition, but in others also, from two to twelve years of age. A variety of gastric affections, with and without pyrexia, occurred in children of all ages; and in some, they terminated in Diarrhœa, Dysentery, and Cholera Morbus.

John B. Davis, } Physicians.

Wm. Shearman, }

To the enlightened Physicians who have drawn up the foregoing Report, the Editor of this Journal begs to return grateful thanks; and hopes for a continuance of their favours.

---

### III.

### *Case of sudden Loss of Sight, &c. with the Appearances on Dissection.* By George Williams, Esq. Surgeon, Portsea.

About two years ago, a boy, ten or twelve years of age, was suddenly stricken totally blind, while sitting writing in a school-room in Portsea. He was led home, in perfect health, in other respects, but incapable of discerning any thing. Nothing was apparent in the eyes themselves, and his health continued good for some months afterwards. Setons, cupping, purgatives, mercurials, &c. were tried, but without producing the least effect. At length he began to complain of head-ache; then his intellects tottered; and lastly, emaciation advanced to the most extreme degree that perhaps ever was seen in the human fabric. For many months, the poor boy was quite idiotic, and never uttered any other word than "Mother!" "Mother!" He died, or rather ceased to exist, about the 20th of July last; and the following were the appearances in the brain, as observed by Mr. Porter and myself.

The skull was a thin one: on holding the calvarium to the light, it appeared unusually diaphanous in many parts. The dura mater was increased in thickness and density, and, from the quantity of fluid beneath it, appeared like an ox bladder distended with water. On raising this membrane, at least eight ounces of a perfectly pellucid serous fluid escaped.

The pia mater was increased in vascularity. From the length of time which had expired before examination, the extreme heat of the weather, and the maceration it had been subjected to in the effused fluid, the substance of the cerebrum was so soft and pulpy as to preclude any thing like a demonstration of its parts. Separating the hemispheres, we saw the corpus callosum rising up into a considerable convexity, in consequence of the distended state of the lateral ventricles, which were so enlarged as to contain nearly three ounces of fluid each. Indeed, all the ventricles were much increased in size from the same cause. It was remarked, that the whole brain appeared more vascular than usual; the blood-vessels on the pons varolii, medulla oblongata, and as far down the medulla spinalis as we could see, were as if injected.

The morbid appearance of the optic nerves was particularly striking. In consequence of the pressure they had been subjected to, not a single nervous fibre could be traced in them; the neurilema thickened and opaque, being all that remained. This appearance commenced a short distance before their bifurcation, and continued to their insertion into the eye-ball.

GEORGE WILLIAMS.

16, St. George's Square, Portsea,
September, 1818.

### IV.

### *Nux Vomica in Paralysis.*

M. BRICHETEAU has drawn up a number of Cases of this disease, treated at the Hotel-Dieu, on the plan of Fouquier; some of which we shall here condense.

*Case* 1. Midi, about forty years of age, was admitted into the Hotel-Dieu the 1st of July, 1816, for the cure of hemiplegia of the right side, succeeding an apoplectic attack. No power of motion in the right arm or leg, nor power of utterance; tongue hanging out of the mouth, and drawn towards the opposite side. A vomit exhibited without operation.—2d July. Four grains of nux vomica in powder. The patient began with doses of two grains, and gradually increased them to four. No inconvenience resulted, and a daily amendment in the complaint was soon observed. When the daily quantum of the medicine amounted to fifty-two grains, the cure was complete.

*Case* 2. Janhin, ætat. 49, an old soldier, had been affected during ten years and more, with rheumatic pains in various parts, but especially the right arm, which, since the commencement of 1816, was completely paralytic. He never had had the venereal disease. He was admitted on the 26th of July, and put on the nux vomica in powder; the daily quantum was gradually augmented to fifty grains, by which time (15th of October) he was completely recovered.

This man frequently complained of a sense of burning heat in the stomach, during the exhibition of the medicine, which induced M. Asselin, the physician, to suspend, for a time, its use. In common also with other patients taking this remedy, he often felt convulsive twitches in the paralysed member, more rarely in other parts of the body.

*Case* 3. Vanhove, a shoe-maker, sixty-two years of age, was received into the Hotel-Dieu the 25th of June, 1815. He reported that the evening before, having felt a giddiness, he fell down in his room, and two hours afterwards, having recovered his senses, he found himself paralytic on the left side. In this state he was when he entered the hospital. He lay on his back, the head spontaneously reclined to one side.

After having fruitlessly tried venesection in the foot and a blister to the neck, he commenced, on the 24th of June, the powder of nux vomica, in doses, at first, of two grains, which was cautiously increased to 22, and afterwards to 64 grains in the day. He had scarcely got to 20 grains a day, when the amendment was astonishing; and by the 1st of August there was hardly a symptom of paralysis. He walked through the ward with the aid of a stick, and used his arms with ease. He took, during several days, half a drachm of the powder by *glyster*, which produced a singular effect, for it brought on the most alarming convulsions, and was thence discontinued. He remains as yet in a state of partial hemiplegia,

This remedy is certainly deserving of a trial in so distressing and intractable a disease.

---

### V.

### *Case of Gastrotomy, with Remarks.* By M. Monfalcon.

Previously to examining how far Gastrotomy is indicated in internal strangulation, M. Monfalcon records a case of this operation, which he had witnessed in one of the large hospitals of Paris.

A healthy man, aged 57, experienced sensations of uneasiness from a moderate repast, and, some days afterwards, slight indigestion, in consequence of having eaten a large quantity of cherries, and swallowed the stones. During the two days which preceded his admission into *l'Hotel-Dieu*, were observed a painful and progressive tumefaction of the abdomen; pain, particularly severe, about the right iliac region; suppression of the alvine discharges; sensible diminution of the temperature of the hands and face; and commencing alteration of the features.

On the day of his admission, July 30th, 1817, he exhibited the following symptoms: A pale shrunk countenance, expressive of much pain; melancholy presentiments; general coldness of the surface, especially of the hands and face; excessive abdominal tension, more strongly marked about the region of the transverse colon; great sensibility, particularly in the right iliac region just above the cœcum; dry mouth; intense thirst; tongne somewhat pasty; bowels obstinately constipated; pulse but little altered. The treatment consisted of copious bleeding from the arm; application of leeches to the anus and the regions of the transverse and descending colon and of the liver; emollient fomentations and liniment to the abdomen.—31st. No sensible alteration. Liniment and fomentations continued; an injection; eighteen leeches in the situation of the ascending colon. On the morrow, the debility was increased; the constipation unsubdued; and the abdominal sensibility more general and exquisite. The pulse was become somewhat feeble and wiry, and the coldness of the hands and face more intense. Cataplasms, in addition to the fomentations and liniment; and application of twenty-eight leeches to the abdomen.—August 2d. Reiterated vomiting of a matter of a golden-yellow colour, and intolerable fecal odour, preceded and followed by eructations, hiccough, and nausea; paleness of the face; oppression and anguish, and momentary change in the seat of the pain, which passed from the right to the left iliac region; and increased smallness and concentration of the pulse. Local remedies continued, with injections of oleum Ricini.—3d. Continued vomiting and constipation, hiccough and nausea; and, at the same time, excessive and painful tension of the abdomen, and icy coldness of the surface and extremities. Conjoined with these symptoms, the state of the pulse, and failure of the remedies hitherto prescribed, led to the proposal of gastrotomy; which, however, was not then performed. On the following morning, the abdomen was re-examined with the most scrupulous attention; and, notwithstanding the loss of a day, the situation of the man appeared to be no worse. The pain, which for two days had passed to the left iliac region, was become, though less intensely, fixed in the right. Upon the seat of the pain, a kind of puffiness and deep-seated fluctuation was discovered. They had existed ever since the admission of the patient into the hospital. The operation was then determined on, and performed in the following manner:

An incision was commenced at the umbilicus, on the linea alba, and carried about three inches and a half downward. The peritoneum was then exposed and cut with all due precaution. The operator, not seeing any intestine present at the wound, inferred the existence of adhesions; introduced the right fore-finger, smeared with cerate, along the posterior surface of the abdomen to the cœcum; and encountered a kind of pouch formed by recent adhesions; on the rupture of which several spoonsfull of a flaky pus, resembling the exhalation of serous membranes, issued from the wound. A slight sinking of the right side of the abdomen ensued. A second pouch was afterwards discovered, and emptied itself like the first; but the surgeon failed in his efforts to detect the seat of the internal strangulation. He could not even draw out externally a knuckle of intestine; and, after having exhausted all the resources of art, and taken the opinion of many able practitioners who were present, he deferred making any further trials till evening. The dressing consisted of a partial reunion of the wound, and of the application of a compress spread with cerate.

During the two first hours which succeeded the operation, the vomiting subsided, but not the hiccough. Soon afterwards, however, the nausea and vomiting re-appeared; the strength sank; the pulse became imperceptible; and the patient died during the night. Upon dissection, on the morning after, membranous adhesions were found existing between the abdominal parietes and the intestine. Deposits of pus, circumscribed by adventitious membrane, were found, here and there, dispersed between the intestines, the liver, and the diaphragm. Almost the whole pelvis was filled by a serous pus, which surrounded the rectum, and had separated it in the greatest portion of its extent. The surface of the small intestines was red, and their calibre considerably augmented; but the large intestines displayed a remarkable contraction. The omentum, verybroad at its origin from the colon, became narrower as it approached the superior aperture of the pelvis, and adhered, in an extent of four or five inches, to the termination of the ileum, till its entrance into the cœcum, to which it was connected, assuming the figure of a goose's foot. This adhesion resembled two open fans, connected by their summit. A portion of the small intestine passed behind the omentum, between this membranous fold and the cœcum, beneath the band, and descended into the pelvic cavity. The whole of the portion above the omentum was enormously distended by gas and fæcal matter; while that immediately below was so contracted as not to equal the volume of the little finger. There the swollen and thickened intestinal parietes presented an oblique prominence, corresponding to the direction of the omentum. Thus had a loop of intestine been strangulated at the angle formed by the omentum and small intestine. The adhesions seemed to have been of very long standing. Dr. Monfalcon observes, that this case of strangulation belongs to his ninth species.

Dr. Monfalcon next proceeds to animadvert upon the manner in which the operation in this instance was performed; and declares himself decidedly hostile to gastrotomy for the removal of internal strangulation, both in principle and practice. To compare it, when instituted for this end, with wounds of the abdomen, lithotomy, or gastrotomy performed in extra-uterine pregnancy is, he contends, to draw a false parallel: for these last operations have a fixed object; their indications are positive; and the nature and causes of the disease are perfectly understood. He then reviews the case of a kind of internal hernia, sometimes since published, with remarks on gastrotomy in internal strangulations, by M. Fages of Montpellier. Here the knuckle of intestine was lodged in a particular sac of the peritoneum, situated on the anterior and middle part of the psoas muscle, and on the right side of the rectum. On dissecting the cellular structure by which the sac was united to these parts, he found the body of the testicle exposed, and a portion of the epididymis at the posterior inferior part of the sac, as though they had been pushed from the tunica vaginalis by the intestine which occupied their place; while the anterior part of the epididymis was in the sac with the intestine. The patient had informed M. Fages that his disease had originated from his having hastily stooped down to pick up something, when he felt within the abdomen, about the right iliac region, a kind of crepitation, which was followed, sometime afterwards, by pain in the part. This pain progressively increased in violence. Monfalcon dissents from M. Fages in thinking that the patient, in this case, would not have been saved by the operation of gastrotomy, however seasonably performed.

The only circumstances under which M. Monfalcon considers this operation warrantable, are the following: a sense of cracking felt by the patient, when in good health, previously to the occurrence of internal strangulation; and the supervention of a violent and fixed pain in the part; the extension of the sensibility from this painful part as from a centre, to the other parts of the abdomen; and, finally, the lapse of but a short interval from the invasion of the inflammatory symptoms. But, on the other hand, when the origin of the symptoms is obscure, or the pain presents not the characters just indicated; when the enteritis has atttained a very high degree of severity; and there exist coldness of the extremities, clammy perspiration on the face, decomposition of the features, and other symptoms of extreme irritation of the abdominal viscera, he hesitates not to reprobate gastrotomy as an absurd and barbarous operation. Even when the conditions are all favourable, the surgeon ought not to take up the knife, till after having investigated, with the utmost care, all the signs indicative of the seat and nature of the internal strangulation. What disgrace and regret must the surgeon experience, who, upon opening the abdomen of the patient, should fail to discover the strangulation, the existence of which he had, just before, confidently predicted.

What then should be the treatment of a patient doomed to destruction by such a disease, M. Monfalcon is unable satisfactorily to point out. But he hesitates not to prefer the efforts of Natnre to the interference of Art. Who will believe, he inquires, that the persons who have recovered, after the discharge of a great length of gangrenous intestine by the anus, would have done equally well under the attempts of a surgeon to destroy the volvulus by the operation of gastrotomy? In every case, he observes, local and general blood-letting, baths, emollient fomentations, and diluents are remedies infinitely preferable to irritating injections, active stimulants, and the different mechanical means, which have too frequently been employed by unfeeling practitioners, to the torture of the unhappy victims of internal strangulation.

### MISCELLANIES.

### *Morbid Anatomy of Hæmorrhoidal Tumours.*

(From the French of LARROQUE.)

1. Larroque, having exposed the erroneous opinions entertained by Hippocrates and Morgagni, relative to the *varicose* nature of hæmorrhoidal tumours, points out two distinct varieties of them, viz. The spongy or cellular, and the encysted. On making an incision through the former, a reddish and uniform parenchymatous structure is developed, from which blood and serum issue on pressure, and which grows pale and flabby when macerated in water. The veins lie between this substance and the common integuments, and exhibit no trace of varicose affection, at the commencement of the disease.

In the second, or encysted variety, there is a smooth white internal surface, when empty, but reddish when filled with blood. These cysts occupy the centres of the tumours, but sometimes lie under their external tunics, separated from them by a dense layer of cellular membrane. When there are dilated veins, they are more intimately connected with the coverings of the tumours than with the cysts. P. 61, *et seq.*

### *Hysteria and Hypochondriasis.*

(From Villermay.)

2. What is the seat or nature of Hysteria? Its seat is in the uterus, as the name expresses. An irritation or spasm is determined on this organ, of which the patients themselves are frequently sensible; and this irritation is totally independent of organic lesion, or alteration of tissue. During a paroxysm of hysteria, if the hand be placed on the hypogastric region, a kind of vermicular motion will be felt there, and the same will be felt if the finger be introduced into the vagina. The *general* phenomena are the result of that sympathetic influence which the nerves of the womb exert on those of every part of the body.

This obscure convulsive movement in the uterus is terminated by a utero-vaginal emission, or rather secretion of mucus, and a profuse discharge of limpid urine, circumstances which would induce us to conclude, that Nature effected a depletion of the vessels in this manner, and thus restored the balance of the circulation and excitability.

The same author considers the seat of hypochondriasis to be in the abdominal viscera, primarily, and especially in the stomach. The vital powers or properties of the abdominal viscera become altered from a variety of causes. Derangements in the function of digestion succeeds, and this derangement is propagated by sympathy or contiguity from organ to organ, till at length the intellectual functions are affected; and these in their turn re-act on the corporeal derangements, and aggravate the original evil.

This we conceive is sound pathology. It points plainly to the proper mode of treatment. Nothingbut a restoration of the abdominal functions to their proper degree of integrity can check the spread of hypochondrial ailments,

### *Pathology of Phthisis; and the Influence of Climate over its Developement.* Broussais.

3. M. Bayle divides Phthisis into the tuberculous, calculous, cancerous, and ulcerous; almost all of which species, however, have the tubercle as the basis of the disease. I have observed, and dissected as many cases of this disease as M. Bayle, and have thence come to nearly similar results. I have only met with a single case of ulcerated lungs unaccompanied by tubercles, or some analogous disease of the lymphatic or cellular structure of the organ. Now it is on the tuberculous degeneration that the physician ought particularly to fix his attention. My researches have led me to conclude, that tubercles in the lungs are the result of, and developed by *irritation* in the sanguiferous capillaries of that organ; which irritation is greatly influenced by climate, as the following facts will shew.

In 1804, I joined the army of Holland, and there found phthisis so prevalent that, whenever a common catarrh came to be protracted among people of fair complexion and lymphatic temperament, phthisis was the termination. I opened every one who died of the disease, and invariably found tubercles or analogous granulations in the lungs. From this circumstance I was led to infer, that had these unfortunate men remained in their native climates, the greater number of them would have escaped that pulmonic irritation and inflammation which developed tubercles, and ultimately induced phthisis. This inference was fully realized soon afterwards. The army was marched to Italy, where pulmonary diseases and phthisis almost entirely disappeared; and what is remarkable, the same classes of soldiers who were every day dying of phthisis in Holland, presented, when dead of any other disease in Italy, not a trace of tuberculous degeneration in the lungs. This sufficiently proves that a cold, moist, and variable climate not only tends to drive tubercles into suppuration, when once formed; but, by keeping up irritation in the respiratory apparatus, actually has the power of producing the tubercles themselves.

4. *Spontaneous Obliteration of the Femoral Artery.* M. Biot operated in the old way on popliteal aneurism. After laying open the tumour seven inches in length, and scooping out several pounds of coagula, the tourniquet was slackened, but no blood was poured forth from the artery. Diligent search was then made, but no trace of the vessel could be found, nor was there any subsequent hæmorrhage.

5. *Death from an Operation.* M. Biot extirpated a large cancer from the breast of a female. The patient experienced such excruciating pain during the first incision, and the whole muscles of the body were thrown into a state of such rigid contraction, that it required six men to hold her. The surgeon was obliged to stop the operation for two minutes, till the spasmodic action ceased; she then appeared as though she had exhausted all her energy, and made no complaint during the sequel of the operation. The tumour removed, no hæmorrhage succeeded. She was placed in bed, the dressings applied, and she appeared indifferent about any thing except repose, which she much wished for. She died in a few minutes!

6. ***Abscesses in the Liver from Contusions and Wounds in different Parts of the Body.*** M. Biot, in his late work on Military Surgery, has made some sensible observations, and related several interesting cases of this pathological phænomenon. Of seven soldiers, who died in the military hospital at Strasbourgh from wounds of the head, in the months of January and February 1794, six shewed abscess in the liver. But it is not from wounds in the head alone that hepatic suppurations are determined. A countryman received a musket ball in the foot at the blockade of Besançon, which fractured several of the tarsal and metatarsal bones. A more than common symptomatic fever seemed to ensue: his skin turned yellow; the right hypochondrium swelled; the belly became tense; and he died on the 12th day from the receipt of the injury. The liver was found in a mass of suppuration and gangrene.

D. Boban relates, that in the month of February, 1809, two artillery-men of his regiment received, at the same time, some violent kicks of a horse on the fore part of their legs, and were conducted immediately to the military hospital. The contusions did not appear to be of any dangerous consequence, but on the 8th day jaundice appeared in both cases. The abdomen became tense and painful, with obstinate constipation, and they both died, one on the 13th, and the other on the 14th day of the accident. In one there was a large abscess in the liver; in the other, several small abscesses and ulcerations in the same viscus; and also an abscess in the lungs. No prevalent disease reigned in the hospital at this time.

M. Larrey relates, that on opening General Caffarelli, who died after an amputation of the left arm, a large abscess was found in the liver, and another in the lungs.

M. Curtet, surgeon of the military hospital at Brussels, after the battle of Waterloo, observed numerous instances of hepatic abscess in those who died of wounds in various parts of the body. (Actes de la Societé de Bruxelles.)

M. Biot pretends not to explain the reason of this connexion between hepatic abscess and external wounds; but conceives, that in a considerable number of these the epigastric region falls into a peculiar state not yet sufficiently understood, which disposes the liver to inflammation and abscess. "Peut-etre dans un assez grand nombre de blessures, le centre epigastrique eprouve-t-il un etat particulier qui n'est pas encore suffisament connu, qui dispose le foie à un etat inflammatoire, &c."

7. *Foreign Bodies in the Œsophagus.* It not unfrequently happens, that we see a fellow creature snatched suddenly from this life by the arrest of a foreign body in one of the passages leading to the lungs or stomach. Although the *latter* is less frequently fatal than the *former*, on account of the possibility, generally speaking, of forcing the foreign body down into the stomach by means of a probang; yet it occasionally happens, that it is either impracticable or imprudent so to do; and then we are at our wit's ends, for to cut down upon the œsophagus is no trifling operation. The two following Cases will not therefore be considered uninteresting or useless, as they elucidate a remedy which we believe has not been employed in this country.

*Case* 1. "A soldier swallowed a piece of tendon of beef, which stuck fast in the middle of the gullet. He was immediately seized with anxiety, convulsions, and fell down on the ground. The surgeons present endeavoured to force down the obstruction into the stomach with a probang, but in vain. The symptoms now became aggravated; the convulsions were uninterrupted; the abdomen became tumefied; the face, the hands, and the feet, grew cold; the voice feeble; a cold sweat covered the whole body; the pulse faltered; and in short, death was at hand! M. Kohler immediately opened a vein in the arm, and injected a solution of ten grains of emetic tartar, which in half an hour brought on so violent a vomiting, that the piece of tendon was thrown to a distance of eight feet, and the poor man was instantly relieved. *Dict. des Sciences Med.* tom. vii. p. 22.

*Case* 2. "A man, 60 years of age, who had no teeth, ate a quantity of beef for supper, without sufficiently chewing it. A piece of it stuck in his throat, and every attempt to push it down was unavailing. The patient was on the very point of suffocation, when Surgeon Knopff dissolved four grains of tartrite of antimony in half an ounce of warm water, which was injected into the median vein of the right arm, at a blood heat, by means of a syringe with a small pipe. In one minute the patient began to feel the effects, and very soon afterwards vomited up a piece of beef as large as an ordinary egg, and was instantly relieved." *Journal Gen. de Med.* tom. xxxii.

8. *Sulphur Vapour Baths.*

Dr. Johnson, the Editor of this Journal, has directed his attention, during the last two or three months, to the effects of the Sulphur Vapour Baths, constructed on Gallé's principles, as erected in Red Lion Square, London. He has seen very considerable advantages derived from their application, in various cutaneous diseases of great obstinacy and inveteracy, and also in paralytic, rheumatic, and chronic arthritic affections. It is merely a hot air bath, in which is suffused the vapour of sulphureous acid, arising from sulphur thrown on a heated iron plate. The air and gas are applied to every part of the patient's surface, except the face, by a very ingenious apparatus, and in one or two minutes he falls into a profuse perspiration. After staying in the bath for twenty minutes, or half an hour, he goes into a warmed bed, where he continues perspiring for an hour or two longer. The temperature of the bath is generally about 120 degrees of Fahrenheit. It promises to be a very powerful remedy, and much more efficacious, in many cases, than liquid baths.

9. *The following Case of Resuscitation was lately communicated to the Royal Humane Society.*

As a knowledge of the means to be resorted to on such important occasions cannot be too widely disseminated, we have great pleasure in submitting the detail to our Readers:—On Monday, May 4th, 1818, James Carney, a boy, aged eleven years, while at play on board a keel or coal lighter, fell into the river Wear. A strong ebb tide forced him under a ship's bottom, where he remained for a short time; but being, by the rapidity of the current, hurried away for nearly half a mile, he must have inevitably perished in the sea, although but a short distance from the harbour's mouth. Providentially, Robert Kirkhouse being in his boat, saw his hat on the furface of the water, and his arm appearing, he laid hold, and pulled him into his boat, to all appearance lifeless. The period of time, from his falling into the water to that of his arrival at the shop of a surgeon, was a full half hour at least; but how long he might have been immersed could not be ascertained. His whole aspect exhibited a state of apparent death; his body stiff and inflexible; his face swollen, and jaw completely locked. He was immediately stripped, put to bed, enveloped in warm blankets, his head reclined on pillows, bladders of hot water applied to his feet, and frictions with flannels, by four persons, to the whole surface of his body, were used, whilst the medical attendant rubbed the region of the heart, stomach, &c. with vol. alkali and camphorated spirits; and on gently pressing it, the air and froth issued from his mouth and nostrils. These methods being persisted in for about twenty minutes longer, his body felt warm, and the limbs became somewhat more flexible, yet a full half hour elapsed before any symptoms of vitality appeared. It was evinced by slight convulsive twitchings of the muscles of the face, accompanied with a fluttering, a palpitation of the heart, and also a gradual disappearance of the lividness of his complexion. His jaws being so far relaxed as to admit of a spoon between his teeth, the surgeon attempted to get down some warm diluted white wine; but this not succeeding, he applied his mouth to that of the patient, and at the same time closing his nostrils, made repeated efforts to inflate the lungs, and using gentle pressure on his chest; the patient then fetched several convulsive sobs. These exertions were continued one hour longer, and when nearly two hours had elapsed, some spoonsfull of diluted white wine were given with advantage. He began to revive; he screamed loud and struggled hard; threw about his arms and legs in such a manner, that it was found difficult to keep him quiet. The pulse at the wrist was scarcely perceptible till about this period, but it afterwards became stronger. He passed a trouble-some night; but, towards the morning, slept pretty well. Some medicine was prescribed, and the next day he seemed quite recovered. He remains in perfect health.

10. *Ligature on the Arteria Innominata.*

Dr. Nelson, of Richmond, Virginia, in a letter to Dr. Seeds, relates the following circumstance:—" Dr. Mott, of New York, has tied the *arteria innominata* for an aneurism of the subclavian artery. I saw the patient six days after the operation; but I regret to add, that I have since heard he died of secondary hæmorrhage, after the ligature came away. However, the practicability of the operation is established beyond doubt."

*Note by the Editor.*—In the "*Nouveau Journal de Medecine*" for July last, this operation is alluded to, with this *trifling* deviation fromtruth. "Vingt neuve jours après, le malade alloit bein; la ligature étoit tombee onze jour après l'operation."

### 11. *Fistulous Wound of the Stomach.*

Professor Lombard relates the case of a man who was wounded by a small-sword, which penetrated the stomach. A fistulous opening kept up a communication between the internal surface of this organ and the external surface of the body, by which channel the patient could, at pleasure, discharge the contents of the stomach, when he found he had eaten too much. What would some of our London Epicures give for such a convenient safety-valve!

### 12. *Poisoning by Oxalic Acid.*

A correspondent suggests the following hints for preventing those calamitous accidents which so frequently occur from oxalic acid being taken by mistake for sulphate of magnesia, or Epsom salts.

"How far the colourless purity of oxalic acid may be required, when used in the large way by calico printers or others, I cannot say; but as the principal object for which it is introduced into private families is for the purpose of cleaning leather, particularly boot-tops, a slight tinge of yellow would be no disadvantage to it. To give it this tinge, preparations of iron would be inadmissible, as they would blacken the leather; but I think an infusion of some of the vegetable yellows, as turmeric, fustick, French berries, or such other substance as will present itself to the skilful chemist, might be employed; and such acid in future as should be kept for retailing, should be ordered and known by the name of the 'Yellow Oxalic acid.' This, I trust, would in some measure obviate such mistakes as have lately occurred."

### 13. *Medical School in Dublin.*

We have been favoured with an account of a complete Medical School in Ireland, which, we are sorry, from the limits and select nature of this Journal, cannot be inserted at full length. We shall, however, endeavour to give a faint outline of it here.——There are six Professorships. The University Professors deliver annually a *public Course of twelve Lectures*, on their respective subjects. Lectures on Anatomy, Surgery, and Chemistry, are delivered, from the beginning of November, till the end of April, in Trinity College. On the Institutes of Medicine, the Practice of Medicine, Materia Medica, and Pharmacy, in Sir P. Dunn's Hospital. On

Botany, from the beginning of May till the end of July, in Trinity College. Terms for each of these Courses of Lectures Four Guineas.—Clinical Lectures are given alternately by the Professors, at least two days in the week; admittance Three Guineas. Anatomical demonstrations are given daily by the Demonstrator of Anatomy in Trinity College. The Students are superintended in their dissections, and subjects are provided for the muscles, blood-vessels, and nerves. A private room for Practitioners. Dissections, Subjects, and Demonstrations Six Guineas.—Lectures are also given on Comparative Anatomy, Cutaneous and Ocular Diseases, Operative Surgery, Chemistry, &c. on reasonable Terms. A Medical Society holds weekly meetings in Trinity College, for the purpose of discussing subjects connected with Medicine, Surgery, and Pharmacy. A Medical Circulating Library, belongs to the Members. Admission to the Society, with the use of the Library, One Pound. Medical Officers of the Army and Navy, admitted to the Surgical and Anatomical Lectures in Trinity College without fee.

In the Hospital, the Clinical cases are recorded—the bodies of patients who die are examined, as often as possible, and the morbid appearances explained to the Students. "All pupils are permitted to attend the entire practice of the Hospital during a year, for Three Guineas."

*The Students who do not graduate in Arts*, are permitted, at the end of three years, from the date of their matriculation, to undergo an examination, write a Thesis in Latin, and if approved, to publish that Thesis. They then receive a public testimonium from Trinity College. Those who go through a Collegiate Course are, three years after having graduated as Bachelor of Arts, permitted, upon proper examination, to take the degree of Bachelor of Medicine; and after passing a second examination, and publishing a Thesis, they graduate as Doctors in Medicine; which degree ranks with the degrees of Bachelor and Doctor of Medicine, obtained in the Universities of Oxford and Cambridge. The certificates of attendance are received as giving standing in other Universities, and as qualifications for Medical Officers in the Army, Navy, and East India service.

Upon the whole, we conceive that this school combines a degree of facility, economy, convenience, and privilege, unequalled in any other University in the British dominions, or on the Continent of Europe.

### 19. *Case of Urticaria.* By D. J. H. Dickson, M. D. &c.

John Bennet, ætat. 5, February 4th, 1818, was yesterday seized suddenly with febrile symptoms, for which he took a dose of calomel and antimonial powder at bed-time, and a saline purgative this morning. He has been freely purged; but the pulse is still quick, and the tongue white.—℞. Liq. ammon. acet. aquæ puræ āā ℥ij; liq. ant. tart; syr. pap. errat. āā ʒij; spt. ætheris nit.

♏ xl M. Capiat cochleare mag. ter. quaterve in die. Rep. hyd. sub. et pulv. ant. āā gr. ij, hora somni. 5th. There is an eruption of wheals on his arms, thighs, belly, and between his shoulders. They are elevated, red, circular, have a white top, and very much resemble the stinging of nettles. He appears heavy; his face, particularly around the eyes, is a good deal swelled; there is head-ache; quickened pulse; white tongue. The bowels are open.—Rep. sub. hyd. H. S. 6th. There was a considerable increase of the eruption, with much heat and itching last evening; which becomes faint and scarcely perceptible in the morning. Pulv. jalap gr. x; calomel gr. iij. 7th. Less swelling of the face, and very little eruption to-day. Was slightly delirious last night; but is sensible to-day. Some head-ache; two black motions.—Infus. sennæ ℥ij. 8th. Some pain in one side of his head; very little fever; face a little swelled; very little eruption last night; two dark motions.—Pulv. jalap gr. xij; sub. hyd. gr. iv. statim; et habeat infus. sennæ ℥j ß. Post Meridiem, si opus sit. 9th. Was freely purged last evening, and again this morning; the motions offensive, like slime or mud, and tenaciously adhere to the bottom of the vessel. Scarcely any eruption, and no fever.—Rep. infus. sennæ. 10th. One costive motion. Urticaria re-appeared last night, with heat and itching on the arms, thighs, and back. Twelve grains of jalap with four of calomel, followed by the senna mixture till the bowels are well cleared. Three grains of calomel at bed-time. 11th. The medicines procured only *one black offensive stool.* The eruption was again very evident last night.—Pulv. jalap gr. x; pulv. scammon. compos. gr. vij statim sumend. et rep. infus. sennæ. P. M. si opus sit. 12th. The eruption was very thick last night; has had several small light-coloured motions. To take the jalap and scammony early to-morrow morning. 13th. Has been frequently purged; the motions, at first, very black, adhesive, and offensive, latterly light, and the last almost white. 14th. No eruption last night, and quite free from fever. 15th. The eruption re-appeared last night partially on the belly and fore-part of the body, with much itching, and swelling of the face; but it has now entirely receded, and he seems very well.—Calomel gr. ij with p. cret. every night or every second night. 16th. The eruption again come out last night. He was again purged, and from this time there was no return of the eruption.

Upon questioning the mother repeatedly, she denied his having taken any improper ingesta, until, on mentioning almonds, she recollected his having received a present of these from the master of a vessel, and eaten very heartily of them.

It is probable, that the foregoing case of Urticaria would have been benefitted by the exhibition of an emetic at the commencement; but from the mother declaring that no improper or unusual food had been taken by the child, and the stomach not being more affected than is usual in febrile complaints, while the intestinal excretions were extremely depraved, my attention was chiefly directed to the bowels, which, as may be seen, required a very frequent and active exhibition of purgatives for so young a patient. In May last, I met with another well-marked case of urticaria in a woman of 40, which occurred after some strenuous exertion, and having overheated herself. It was attended with considerable fever, and intolerable itching. I expected it would have proved obstinate; but it gave way to two or three active purgatives.

D. J. H. Dickson.

*Clifton*, 1818.

The preceding case affords an excellent specimen of what Dr. Wilson would term "a morbid sympathy," between the digestive organs and the surface of the body. It also shows to what extent purgatives may, and indeed must be, carried in infantile disorders. In them, the digestive organs, the liver, and the brain, are the great foci of irritation and disease, and from *these* three series of parts we must manfully deplete in all acute affections. Ed.

### 20. *Injury of the Head, followed by Suppuration within the Cranium.*

A boy was admitted into a public hospital in the metropolis, with a wound on the anterior part of the head, about two inches in length. He was perfectly sensible; complained of but little pain, and that only in the direction of the wound, and chiefly when touched. The scalp appeared much bruised. The wound was dressed simply with adhesive straps. Leeches were applied to the temples, and the bowels well opened, by which means he was so far relieved as to be made an out-patient in the course of four or five days. About a fortnight afterwards he was again brought back to the hospital by his father, who gave the following account. The boy appeared to be going on well till within two days of his return, when he began to complain of darting pains in the forehead, and in the direction of the coronal suture. About twelve hours afterwards erysipelas began to appear in the face and eyelids of the right side, which parts were much swollen on his re-admission. During the night he had been delirious at intervals. On examining the wound, a considerable collection of pus was discovered beneath the scalp, which was immediately let out, and a poultice applied. Leeches were also put to the cheek and temples; the bowels were kept open. No appetite; delirium during the night. On the second day erysipelas was so much increased, that the right eye was completely concealed, and the left cheek greatly inflamed. The suppuration in the wound augmented; delirium constant; antiphlogistic plan continued. Third day, the symptoms all aggravated; the face swollen enormously; constant, low, muttering delirium; nearly insensible. Several sinuses were discovered between the scalp and the os frontis, from whence was discharged a great quantity of fœtid pus. Considerable hæmorrhage from branches of the temporal artery followed the laying open of these sinuses, requiring the ligature. From this time he sunk gradually, and expired at one o'clock, A. M. the following morning.

*Dissection.* Pus was found extravasated in various directions between the scalp and cranium, from which last the pericranium was detached all round the wound, and the bone of a greenish colour. On removing the calvarium, pus was found between the skull and dura mater; also between this last and the pia mater. The vessels were gorged with blood. In the thorax two or three tubercles were found in a state approaching to suppuration.

This case shews the danger of that *secondary* inflammation which so often follows blows on the head; and affords us a lesson for cautiously watching a patient for a considerable period after the accident; enjoining low diet, open bowels, &c. W.

---

J. C. Carpue, F. R. S. will commence his Anatomical Lectures on Thursday, October 1, 1818, at his Theatre, No. 50, Dean Street, Soho.—The Structure of the Human Body will be explained, as also its Physiology. The Operations of Surgery will be shewn on the dead Body.—Every day some Part of the Human Body is demonstrated to the Pupils, who are required to demonstrate in their turn *(this Plan obliges Mr. C. to limit the number of his Pupils)*—Myology is not taught till the Pupils are perfectly instructed in Osteology, &c. A general Examination takes place every tenth day; and if the Pupils do not perfectly recollect the Parts that have been lectured on, they are again demonstrated.—The Dissecting Room will be open from Seven o'clock in the morning till Five in the evening. The Pupils are here taught the Method of Operating, as also the Art of injecting and making Preparations. Three Courses are given every year.—Further Particulars may be known by applying to Mr. Carpue, 72, Dean Street, Soho.

Dr. Clutterbuck will begin his Autumn Course of Lectures on the Theory and Practice of Physic, Materia Medica, and Chemistry, on Friday, October 2, at Ten o'clock in the morning.—Particulars may be known on Inquiry at his house, No. 1, in the Crescent, New Bridge Street, Blackfriars; or, at the General Dispensary, Aldersgate Street.

Dr. George Gilbert Currey, Fellow of the Royal College of Physicians, and Senior Physician to St. Thomas's Hospital, will commence his usual Course of Lectures on the Theory and Practice of Medicine and Materia Medica, on Friday October the Second.

Dr. George Gregory and Dr. Cloves will commence their first Course of Lectures on the Theory and Practice of Physic, on Wednesday, October 7, at Nine o'clock in the morning, at No. 14, Old Burlington Street,. The Lectures will be continued on Mondays, Wednesdays, and Fridays, at the same Place and Hour, and will terminate towards the middle of January.—Pupils are admitted to attend the Medical, Surgical, and Midwifery Practice of St. George's and St. James's Dispensary, No. 14, Old Burlington Street. The hours of attendance of the Medical Officers are between Eleven and One in the forenoon, every day; at which time they will be ready to give any further Information on the subject that may be desired.

Mr. Guthrie, Deputy Inspector of Military Hospitals, will commence his Winter Course of Lectures on Surgery, on Monday the 5th of October, at five minutes past eight in the evening, in the Waiting Room of the Royal Westminster Infirmary for Diseases of the Eye, Mary-le-bone Street, Piccadilly. To be continued on Mondays, Wednesdays, and Fridays.—Two Courses will be delivered during the season. In each Course the principles of Surgery will be explained, and the practice resulting from them, with reference both to domestic and military Surgery, fully pointed out. The diseases of the Eye, although forming an integral part of the Lectures on Surgery, will, for the convenience of illustration, be delivered every Thursday evening, until completed. The operations referred to in the Lectures will be shewn in each Course. Terms of attendance—Perpetual, Five Guineas; Single Course, Three Guineas.—Medical Officers of the Navy, the Army, and the Ordnance, will be admitted gratis, on obtaining a recommendation from the Heads of their respective departments, which must be presented to Mr. Guthrie between the hours of two and half-past four, at his house, No. 2, Berkeley Street, Berkeley Square.

Dr. P. Mere Latham, and Dr. Southey, will begin their Lectures on the Practice of Physic and the Materia Medica, the first week in October, at the Middlesex Hospital.

The Lectures in the School of Anatomy and Surgery, Great Windmill Street, begin on the 1st of October, at two o'clock.

We feel happy to announce to Gentlemen of the Profession, that a Medical, Chemical, and Philosophical Library, under the management of Mr. T. Burgess, is just established at the west end of the town, No. 55, Great Windmill Street, Haymarket; and we have no doubt will prove a valuable accommodation to the Faculty, as all works relating to medical, chemical, and general science, may be obtained by the single volume, month, quarter, or year. An accommodation of this kind has long been much required, particularly by Gentlemen who remain in town but a short time; we therefore trust it will meet encouragement.

---

### BOOKS IN THE PRESS.

Dr. Armstrong is preparing new editions, considerably improved, of his three works on Scarlet Fever, &c. Typhus Fever, and Puerperal Fever.

Dr. Ayre, of Hull, is about to publish Practical Observations on the Nature and Treatment of those Disorders which may be strictly denominated bilious.

John Ayrton Paris, M. D. F. L. S. Fellow of the Royal College of Physicians, London; Member of the Royal Medical Society of Edinburgh, &c. has in the press, and speedily will be published, in 8vo. the third edition, considerably improved, of Pharmacologia; or, the History of Medicinal Substances; especially as it regards their chemical habitudes and relations; with a view to render the art of prescribing and composing extemporaneous Formulæ less empirical and more successful, by the establishing it upon fixed and scientific principles. Illustrated by Formulæ, in which the intention of every element is designated by key-letters.

R. W. Bamfield, late Surgeon of the Belliqueux and Warrior, ships of the line, serving in the East and West Indies, and author of an Essay on Hemeralopia, or Night Blindness, has in the press, and speedily will be published, A Practical Treatise on Tropical Dysentery, more particularly as it occurs in the East Indies, illustrated by Cases and Dissections.

Dr. Bostock will shortly publish the History and present State of Galvanism.

The second edition of the Elements of Conchology, according to the Linnæan System, illustrated by 28 plates drawn from Nature, by the Rev. E. J. Burrow, A.M. F.R.S. F.L.S. and Mem. Geol. Soc.

Dr. Henry is printing a new and improved edition of his Elements of Chemistry.

A new edition of A. P. Wilson Philip's Work on the Vital Functions, 8vo.

In a few days will be published, Practical Instructions on the Treatment of apparent Death, arising from noxious Vapours, Drowning, extreme Heat or Cold, Infants apparently Still-born, Persons poisoned, and on the preventive Treatment of Persons bitten by Mad Dogs, &c. &c. With Observations on these Accidents, and on the signs which distinguish actual death from that which is only apparent. Translated from the French of Antoine Portal, Professor of Medicine, Anatomy, &c. &c. &c.

Sir Gilbert Blane, Bart. has a work in the press on Medical Logic.

